# Genome wide association study reveals plant loci controlling heritability of the rhizosphere microbiome

**DOI:** 10.1038/s41396-021-00993-z

**Published:** 2021-05-12

**Authors:** Siwen Deng, Daniel F. Caddell, Gen Xu, Lindsay Dahlen, Lorenzo Washington, Jinliang Yang, Devin Coleman-Derr

**Affiliations:** 1 Department of Plant and Microbial Biology, University of California, Berkeley, CA USA; 2 Plant Gene Expression Center, USDA-ARS, Albany, CA USA; 3 Department of Agronomy and Horticulture, University of Nebraska-Lincoln, Lincoln, NE USA; 4 Center for Plant Science Innovation, University of Nebraska-Lincoln, Lincoln, NE USA; 5 Present Address: Department of Plant Sciences, University of California, Davis, CA USA

**Keywords:** Agricultural genetics, Plant ecology, Soil microbiology

## Abstract

Host genetics has recently been shown to be a driver of plant microbiome composition. However, identifying the underlying genetic loci controlling microbial selection remains challenging. Genome-wide association studies (GWAS) represent a potentially powerful, unbiased method to identify microbes sensitive to the host genotype and to connect them with the genetic loci that influence their colonization. Here, we conducted a population-level microbiome analysis of the rhizospheres of 200 sorghum genotypes. Using 16S rRNA amplicon sequencing, we identify rhizosphere-associated bacteria exhibiting heritable associations with plant genotype, and identify significant overlap between these lineages and heritable taxa recently identified in maize. Furthermore, we demonstrate that GWAS can identify host loci that correlate with the abundance of specific subsets of the rhizosphere microbiome. Finally, we demonstrate that these results can be used to predict rhizosphere microbiome structure for an independent panel of sorghum genotypes based solely on knowledge of host genotypic information.

## Introduction

Recent work has shown that root-associated microbial communities are in part shaped by host genetics [1–4]. A study comparing the root microbiomes of a broad range of cereal crops has demonstrated a strong correlation between host genetic differences and microbiome composition [4], suggesting that a subset of the plant microbiome may be influenced by host genotype across a range of plant hosts. In maize, these genotype-sensitive, or “heritable”, microbes are phylogenetically clustered within specific taxonomic groups [5]; however, it is unclear whether the increased genotypic sensitivity in these lineages is unique to the maize microbiome or is common to other plant hosts as well.

Despite consistent evidence of the interaction between host genetics and plant microbiome composition, identifying specific genetic elements driving host-genotype dependent microbiome acquisition and assembly in plants remains a challenge. Recent efforts guided by a priori hypotheses of gene involvement have begun to dissect the impact of individual genes on microbiome composition [6, 7]. However, these studies are limited to a small fraction of plant genes predicted to function in microbiome-related processes. In addition, many plant traits expected to impact microbiome composition and activity, such as root exudation [8] and root system architecture [9], are inherently complex and potentially governed by a very large number of genes. For these reasons, there is a need for alternative large-scale and unbiased methods for identifying the genes that regulate the host-mediated selection of the microbiome.

Genome-wide association studies (GWAS) represent a powerful approach to map loci that are associated with complex traits in a genetically diverse population. Though pioneered for use in human genetics, to date the majority of GWAS have been conducted in plants [10], and it has become an increasingly popular tool for studying the genetic basis of natural variation and traits of agricultural importance. When inbred lines are available, GWAS can be particularly useful; once genotyped, these lines can be phenotyped multiple times, making it possible to study many different traits in many different environments [11]. While GWAS is typically used in the context of a single quantitative phenotypic trait, analyses of multivariate molecular traits, such as transcriptomic or metabolomic data, have also been conducted [12, 13]. More recently, several attempts have been made to use host-associated microbiome census data as an input to GWAS, which in theory will allow for the identification of host genetic loci controlling microbiome composition [14, 15].

In plants, a recent study in *Arabidopsis thaliana* used phyllosphere microbial community data as the phenotypic trait in a GWAS to demonstrate that plant loci responsible for defense and cell wall integrity affect microbial community variation [16]. Several other recent phyllosphere studies performed GWAS to identify genetic factors controlling microbiome associations with mixed degrees of success [16–18]. Compared to studies of the phyllosphere, GWAS on Arabidopsis root microbes identified host SNPs associated within and surrounding genes with characterized roles in immunity, cell-wall integrity, and development [19]. Previous work comparing the root microbiomes of diverse cereal crops has offered conflicting evidence as to whether host genotypic distance correlates most strongly with microbial community distance within root endospheres or rhizospheres [3, 4]. These data suggest that the sample type exhibiting the strongest correlation between genotype and microbiome composition may differ for each host and that an initial evaluation of the degree of correlation between genotype and microbiome phenotype across sample types may be informative. However, to our knowledge, the use of GWAS in conjunction with the rhizosphere microbiome has yet to be explored.

In the context of the root and rhizosphere, we propose *Sorghum bicolor* (L.) as an ideal plant system for GWAS-based dissection of host-genetic control of microbiome composition. Sorghum is a heavy producer of root exudates [20], and the sorghum microbiome has been shown to house an unusually large number of host-specific microbes [4]. In addition, there is a wide range of natural adaptation in traditional sorghum varieties from across Africa and Asia, and a collection of breeding lines generated from U.S. sorghum breeding programs, both of which provide a rich source of phenotypic and genotypic variation [21]. Several genome sequences of sorghum varieties have been completed, and variation in nucleotide diversity, linkage disequilibrium (LD), and recombination rates across the genome has been quantified [22], providing an understanding of the genomic patterns of diversification in sorghum. Finally, sorghum is an important cereal crop grown throughout the world as a food, feedstock, and biofuel, enabling direct integration of resulting discoveries into an agriculturally relevant system.

In this study, we dissect the host-genetic control of bacterial microbiome composition in the sorghum rhizosphere. Using 16S rRNA sequencing, we profiled the microbiome of a panel of 200 diverse genotypes of field-grown sorghum. We test the hypothesis that a subset of the sorghum microbiome responds to the host genotype and demonstrate that this subset shares considerable overlap with lineages shown to be susceptible to host genetic control in another plant host. In addition, we tested whether GWAS can be used to identify specific genetic loci within the host genome that are correlated with the abundance of specific heritable lineages and whether differences in microbiome composition can be predicted solely from genotypic information. Collectively, this work demonstrates the utility of GWAS for analyzing host-mediated control of rhizosphere microbiome phenotypes.

## Methods

### Germplasm selection

**Fig. 1:**
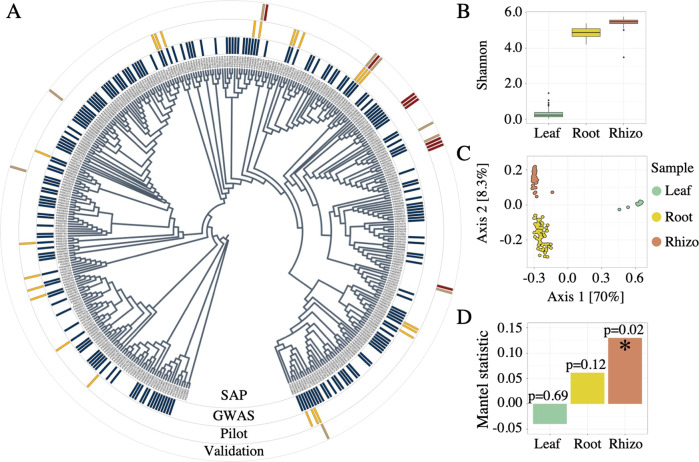
Sample type and population selection. **A** Phylogenetic tree representing the 378 member sorghum association panel (SAP, inner ring), the subset of 200 lines selected for GWAS (second ring from the center, in blue), the 24 lines used for sample type selection (Pilot, third ring from the center, in yellow), and the 18 genotypes used for GWAS validation containing either the Chromosome 4 minor allele (red) or major allele (brown) identified by GWAS (outer ring). **B** Shannon’s entropy values from 16S rRNA amplicon datasets for the leaf (green), root (yellow), and rhizosphere (red) sample types across all 24 genotypes used in the pilot experiment. **C** Principal coordinate analysis generated using Bray–Curtis distance for the 24 genotypes across leaf (green), root (yellow), and rhizosphere (red). **D** Mantel’s R statistic plotted for each sample type across all 24 genotypes indicating the degree of correlation between host genotypic distance and microbiome distance.

In order to ensure that microbiome profiling was performed on a representative subset of the broad genetic diversity present in the 378 members sorghum association panel (SAP) [21, 22], subsets of 200 genotypes were randomly sampled from the SAP 10,000 times and an aggregate nucleotide diversity score was calculated for each using the R package “PopGenome” [23]. From these data, the subset of 200 lines with the maximum diversity value was selected (Fig. 1a, Supplemental Fig. 1, Supplemental Table 1). For the pilot experiment that was used to determine the appropriate sample type for GWAS, a subset of 24 lines was selected that included genotypes from a wide range of phylogenetic distances (Fig. 1a, Supplemental Table 1). The phylogenetic tree of sorghum accessions was generated by the neighbor-joining method using an identity by state (IBS) distance matrix calculated in TASSEL 5.0 [24] and visualized using the online tool: interactive tree of life (iTOL) v5 (ref. [25]).

### Field experimental design and root microbiome sample collection

The experimental field used in this study is an agricultural field site located in Albany, California (37.8864 °N, 122.2982 °W), characterized by a silty loam soil with pH 5.2 [4]. Germplasm for the US SAP panel used in this study [21] was obtained from GRIN (www.ars-grin.gov). To ensure a uniform starting soil microbiome for all sorghum seedlings and to control their planting density, seeds were first sown into a thoroughly homogenized field soil mix in a growth chamber with controlled environmental factors (25 °C, 16 h photoperiods) followed by transplantation to the field site. To prepare the soil for seed germination, 0.54 cubic meters of soil was collected at a depth of 0–20 cm from the field site subsequently used for planting, and homogenized by separately mixing four equally sized batches with irrigation water in a sterilized cement mixer followed by manual homogenization on a sterilized tarp surface. The soil was then transferred to sterilized 72-cell plant trays. To prepare seeds for planting, seeds were surface-sterilized through soaking 10 min in 10% bleach +0.1% Tween-20, followed by four washes in sterile water. Following planting, sorghum seedlings were watered with ~5 ml of water using a mist nozzle every 24 h for the first 3 days, and bottom watered every three days until the 12th day, then transplanted to the field.

The field consisted of three replicate blocks, with each block containing 200 plots for each of 200 selected genotypes. Six healthy sorghum seedlings of each genotype were transplanted to their respective plots, separated by 15.2 cm, and thinning to three seedlings per plot was performed at two weeks post transplanting. Plots were organized in an alternating pattern with respect to the irrigation line to maximize the distance between each plant (Supplemental Fig. 2). Plants were watered for 1 h, three times per week, using drip irrigation with 1.89 L/h rate flow emitters. Manual weeding was performed three times per week throughout the growing season. To ensure that the genotypes were at a similar stage of development and that the host-associated microbiome had sufficient time to develop, collection of plant-associated samples was performed nine weeks post-germination. Only the middle plant within each plot was harvested to help mitigate potential confounding plant-plant interaction effects resulting from contact with roots from neighboring plants of other genotypes. Rhizosphere, leaf, and root samples were collected as described in detail previously [26].

### DNA extraction, PCR amplification, and Illumina sequencing

DNA extractions, PCR amplification of the V3–V4 region of the 16S rRNA gene, and amplicon pooling were performed as described in detail previously [26]. In brief, DNA extractions for all samples were performed using extraction kits (MoBio PowerSoil DNA Isolation Kit, MoBio Inc., Carlsbad, CA) following the manufacturer’s protocol. Amplification of the V3–V4 region of the 16S rRNA gene was performed using dual-indexed 16s rRNA Illumina iTags primers 341F (5′–CCTACGGGNBGCASCAG–3′) and 785R (5′–GACTACNVGGGTATCTAATCC–3′). An aliquot of the pooled amplicons was diluted to 10 nM in 30 μL total volume before submitting to the QB3 Vincent J. Coates Genomics Sequencing Laboratory facility at the University of California, Berkeley for sequencing using Illumina Miseq 300 bp pair-end with v3 chemistry. Sequences were returned demultiplexed, with adaptors removed.

### Amplicon sequence processing, OTU classification, and taxonomic assignment

Sequencing data were analyzed using the iTagger pipeline to obtain OTUs [27]. In brief, after filtering 81,416,218 16S rRNA raw reads for known contaminants (Illumina adapter sequence and PhiX), primer sequences were trimmed from the 5′ ends of both forward and reverse reads. Low-quality bases were trimmed from the 3′ ends prior to assembly of forward and reverse reads with FLASH [28]. The remaining 66,524,451 high-quality merged reads were clustered with simultaneous chimera removal using UPARSE [29]. After clustering, 37,867,921 read counts mapped to operational taxonomic units (OTUs) at 97% identity (Supplemental Table 2). Taxonomies were assigned to each OTU using the RDP Naïve Bayesian Classifier with custom reference databases [30]. For the 16S rRNA V3–V4 data, this database was compiled from the May 2013 version of the GreenGenes 16S database v13, trimmed to the V3–V4 region. After taxonomies were assigned to each OTU, OTUs were discarded if they were not assigned a Kingdom level RDP classification score of at least 0.5, or if they were not assigned to Kingdom Bacteria, which yielded 10,006 OTUs. In the downstream analyses, we removed low abundance OTUs (<3 reads in at least 20% of the samples) because in many cases they are artifacts generated through the sequencing process [31]. Samples with low read counts were also removed. To adjust for differences in sequencing depth and fit a normal distribution, samples for heritability and GWAS analyses were normalized by cumulative sum scaling [32]. For all other analyses, samples were rarefied to an even read depth of 18,000 reads per sample.

### Estimates of broad-sense heritability of OTU abundance in rhizosphere

To calculate the broad-sense heritability (*H*^2^) for individual OTU abundances, we fit the following linear mixed model to OTU abundances of each individual OTU (*n* = 1189) following a cumulative sum scaling [32] normalization procedure that adjusted for differences in sequencing depth and fit a normal distribution:

$$\qquad \qquad {{Y}}_{{\mathrm{ijk}}} = {{u}} + {\mathrm{Gi}} + {\mathrm{Bjk}} + {{e}}$$

In this model for a given OTU, *Y*_ijk_ denotes the OTU abundance of the *i*th genotype evaluated in the kth block of the *j*th replicate; *u* denotes the overall mean; Gi is the random effect of the *i*th genotype; Bjk is the random effect of the *j*th replicate nested within the *k*th block; *e* denotes the residual error. With such a model, we divided the environmental variance by the number of replicates according to [33]. In order to model the spatial trends in the field, we used the 2-dimensional splines approach that was proposed to accommodate the field’s spatial effects by [34, 35]. This approach was implemented in the R package “sommer” [36] that we used for the model fitting. The variance explained by the spatial effects was excluded from the environmental variance for heritability calculation. To get the null distribution of *H*^2^, OTU abundances were randomly shuffled 1000 times and then fitted to the same model as described above. Permutation *p*-value was calculated as the probability the permuted *H*^2^ values were bigger than the observed *H*^2^ value.

### Comparative analysis of heritable taxa between sorghum and maize datasets

To identify the degree to which highly heritable taxa were shared between maize and sorghum, we compared the top 100 most heritable OTUs reported from both maize datasets (referred to as NAM 2010 and NAM 2015) and the sorghum dataset generated in this study. This cutoff, which resulted in a more stringent cutoff than *H*^2^ > 0.15, was used for this meta-analysis because *H*^2^ varied widely between NAM 2010 and NAM 2015, and a single absolute *H*^2^ cutoff would otherwise bias the number of reported highly heritable OTUs between studies. For this analysis, the reported OTUs were aggregated at the order level, resulting in a combined dataset of 300 OTUs spanning 65 bacterial orders. The order level was selected for the following reasons. First, primer differences between studies (V4 in maize compared with V3–V4 in this study), will impact both phylogenetic assignment and resolution at lower taxonomic ranks [37]. Second, taxonomic classification below order was not available for all OTUs. For example, only 14/300 OTUs (4.7%) were not classified beyond phylum or class, while an additional 92 out of the remaining 286 OTUs (32.2%) were not classified below order (Supplemental Table 3). Lastly, when orders were separated into lower classifications, lineage membership sizes were insufficient for downstream statistical analyses.

A subset of the orders (*n* = 18) containing highly heritable OTUs in the maize dataset was not detected in either the high or lowly heritable fractions of the sorghum dataset and was excluded from subsequent comparative analyses. Of the remaining bacterial orders represented by these highly heritable OTUs, we determined the number (*n* = 26) that contained highly heritable OTUs in at least two of the datasets, and the number (*n* = 15) that contained highly heritable OTUs in all three datasets. To understand the taxonomic diversity contained within these 65 orders, the family and genus classification of the 300 heritable OTUs are provided in Supplemental Table 3. To evaluate whether the degree of overlap in highly heritable lineages is greater than what would be expected by chance, we performed a permutation test (*n* = 10,000) in which we resampled 100 random OTUs from the 1189 total sorghum OTUs and recomputed intersections with the two maize datasets. These resamplings were not based on OTU abundance; as such, it would be equally likely to draw an abundant or rare OTU, which avoids the possible confounding issue of heritability being correlated with abundance. *P*-values are reported as the number of instances that these permutations returned a greater degree of overlap in these permutations divided by the total number of permutations.

### GWAS

A genome-wide SNP map of sorghum SAP accessions used in this study was obtained from the community resource generated previously by genotyping-by-sequencing and included characterization at 265,487 SNPs [22]. GWAS was performed using SNPs with a minor allele frequency (MAF) ≥ 0.01 following [38]. For each OTU, GWAS was conducted separately using the best linear unbiased predictors (BLUPs) obtained from the linear mixed model. Population structure was accounted for using statistical methods that allow us to detect both population structure (*Q*) and relative kinship (*K*) to control spurious association. The *Q* model (*y* = *Sα* + *Qν* + *e*), the *K* model (*y* = *Sα* + *Zu* + *e*), and the *Q* + *K* model (*y* = *Xβ* + *Sα* + *Qν* + *Zu* + *e*) described previously [39], were used in our study. In the model equations, *y* is a vector of phenotypic observation; *α* is a vector of allelic effects; *e* is a vector of residual effects; *ν* is a vector of population effects; *β* is a vector of fixed effects other than allelic or population group effects; *u* is a vector of polygenic background effects; *Q* is the matrix relating *y* to *ν*; and *X*, *S*, and *Z* are incidence matrices of 1 s and 0 s relating *y* to *β*, *α*, and *u*, respectively. To account for the population structure and genetic relatedness, the first three principal components (PCs) and kinship matrix were calculated using the SNPs obtained from [22] and fitted into the MLM-based GWAS pipeline for each OTU using GEMMA [40].

### GWAS validation experiment

For the GWAS validation experiment, the 378 genotypes of the SAP were the first subset into lines containing the major (*n* = 343) and minor (*n* = 14) allele for the two haplotypes found at the peak on chromosome 4 described in the text. Including the 178 genotypes not selected for the GWAS, a total of nine sorghum genotypes belonging to the minor allele were selected, with an effort to include genotypes spanning the phylogenetic tree. For each of these nine minor allele lines, another genotype containing the major allele with close overall genetic relatedness was selected, resulting in nine major and nine minor allele-containing lines. Two replicates of each line were grown in growth chambers (33 °C/28 °C, 16 h light/8 h dark, 60% humidity) in a 10% vermiculite/90% calcined clay mixture rinsed with a soil wash prepared from a 2:1 ratio of field soil to water from the field site used in the GWAS. Plants were watered daily with approximately 5 ml of autoclaved Milli-Q water using a spray bottle for the first 3 days, followed by top watering with 15 ml of water every three days. An additional misting was performed to the soil surface every 24 h to prevent drying. Following two weeks of growth, plants were harvested and rhizosphere microbiomes extracted as described for the field experiment.

### Microbiome statistical analyses

All statistical analyses of the amplicon datasets were performed in R using the normalized reduced dataset unless stated otherwise. For alpha-diversity measurement, Shannon’s Diversity was calculated with the diversity function in the R package vegan [41]. Principal coordinate analyses were performed with the function pcoa in the R package ape [42], using the Bray–Curtis distance obtained from the function vegdist in the R package vegan [41]. Mantel’s tests were used to determine the correlation between host phylogenetic distances and microbiome distances using the mantel function in the R package vegan [41] with 9999 permutations, and using Spearman’s correlations to reduce the effect of outliers. Indicator species analyses were performed using the function indval in the R package labdsv [43], with *p*-values based on permutation tests run with 10,000 permutations. Multiple testing corrections were performed with an FDR of 0.05 using the p.adjust function in the base R package stats. Canonical analysis of principal coordinates (CAP) was performed using the capscale function in the R package vegan [41]; an ANOVA like permutation test using the sum of all constrained eigenvalues was performed to determine the percent variance explained by each factor using the function anova.cca in the R package vegan [41].

### Analysis of sorghum RNA-seq datasets

Publicly available sorghum RNA-Seq data for 27 annotated genes in the 1.15 Mb interval of chromosome 4 (Sobic.004G153000–Sobic.004G155900), were downloaded from phytozome v12.1 (ref. [44]). Expression datasets were broadly grouped based on the tissue type from which they were derived (root, leaf, or reproductive). To aid in the visualization of tissue-specific expression of genes exhibiting large differences in absolute levels of gene expression, we normalized the fragments per kilobase of transcript per million mapped reads (FPKM) values for each gene in each tissue type by dividing by the average value of gene expression for that gene across all tissue types. We defined root-specific expression as genes that had a normalized FPKM less than 1 in no more than two root datasets, and a normalized FPKM greater than 1 in no more than two datasets of other tissue types.

## Results

### Diverse sorghum germplasm show rhizosphere is ideal for microbiome-based GWAS

In this study, the relationship between host genotype and microbiome composition was explored through a field experiment involving 200 genotypes selected from the SAP germplasm collection [21, 22] (Supplemental Table 1). While a recent study in Arabidopsis successfully performed GWAS using the root microbiome (endosphere) [19], it did not evaluate microbes that are closely associated with the exterior of the root (rhizosphere). We first sought to determine whether leaf, root endosphere, or rhizosphere samples were most suitable for downstream GWAS in sorghum. Using a subset of 24 genotypes from our collection of 200 (Fig. 1a, Supplemental Table 1), the microbiome composition of leaf, root, and rhizosphere sample types were analyzed using paired-end sequencing of the V3–V4 region of the ribosomal 16S rRNA. The resulting dataset demonstrated comparatively high levels of microbial diversity within both root and rhizosphere samples (Fig. 1b) and strong clustering of above and below ground sample types (Fig. 1c). Three independent Mantel’s tests (9999 permutations) were used to evaluate the degree of correlation between host genotypic distance and microbiome composition for leaf, root, and rhizosphere sample types (Fig. 1d); of the three compartments, the only rhizosphere exhibited a significant Mantel’s correlation (*R*^2^ = 0.13, Df = 1, *p* = 0.02). Based on these results, subsequent investigation of the microbiomes of the full panel of 200 lines, including heritability and GWAS analyses, was performed using rhizosphere samples.

To investigate host genotype-dependent variation in the sorghum rhizosphere microbiome, the rhizospheres of 600 field-grown plants (three replicates of each of the 200 genotypes) were profiled using V3–V4 16S rRNA amplicon sequencing. The resulting data set included 1189 OTUs representing 29 bacterial phyla. Compositional analysis of the microbiome dataset exhibited profiles consistent with recent microbiome studies involving the sorghum rhizosphere [4, 45, 46] from a variety of field sites, with proteobacteria, actinobacteria, and acidobacteria comprising the top three dominant phyla (Supplemental Fig. 3).

### Sorghum and maize rhizospheres exhibit strong overlap in heritable taxa

A recent study of two separate maize microbiome datasets suggests that specific bacterial lineages are more sensitive to the effect of host genotypes than others [5]. To determine if a bacterial lineage’s responsiveness to host genetics is a trait conserved across different plant hosts, the broad-sense heritability (*H*^2^) of individual OTUs in our sorghum dataset was evaluated; *H*^2^ quantifies the proportion of variance that is explained by genetic rather than environmental effects. More generally, this approach treats bacterial abundance as a continuously varying phenotype, similar to plant height, biomass, and yield [47]. In our study, *H*^2^ ranged from 0 to 66% for individual OTUs (Supplemental Table 4). By comparison, *H*^2^ for individual OTUs in the first of two experiments across 27 maize inbred lines had a maximum of 23% (performed in 2010), while the second exhibited a maximum of 54% (performed in 2015) [5]. Further, we used the sorghum diversity panel kinship matrix to calculate the SNP-based narrow-sense heritability (*h*^2^). Consistent with our expectation, the *h*^2^ was lower than the *H*^2^ estimated from the phenotypic data, likely due to overcorrection of the spatial components in the spline analysis. Nevertheless, *h*^2^ was significantly (*R* = 0.34, *p* = 2.2e−16) correlated with previous phenotype-based estimates (Supplemental Fig. 4a), especially for the top 100 heritable OTUs (*R* = 0.5, *p* = 1e−7, Supplemental Fig. 4b).

**Fig. 2:**
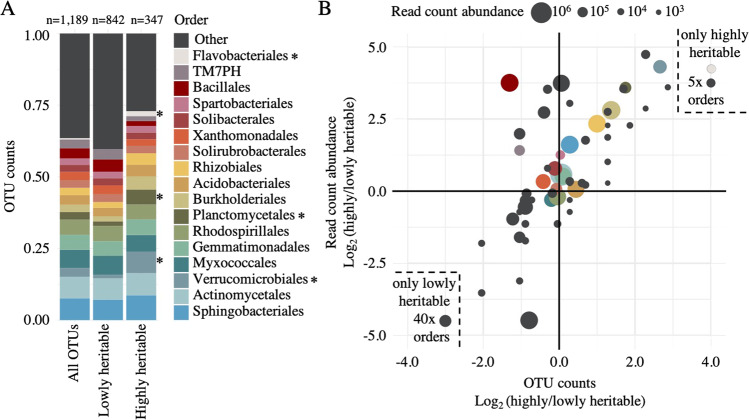
Heritable bacteria are abundant members of the sorghum rhizosphere. **A** The relative percentage of total OTUs belonging to each of the top 17 bacterial orders for all OTUs (left bar), lowly heritable OTUs (middle bar), or highly heritable OTUs (right bar). Orders with significantly different numbers of OTUs in the highly heritable (*H*^2^ > 0.15) as compared to the lowly heritable fraction (*H*^2^ < 0.15), as determined by Fisher’s exact test (*q* < 0.05), are indicated with asterisks. **B** Order-level scatterplot of the log_2_ ratio between highly and lowly heritable OTU counts (*x*-axis) and read count abundance (*y*-axis). Circle sizes represent the total read count abundance of each bacterial order. Bacterial taxa that were present only in the highly heritable (upper right, Flavobacteriales and 5 “other” merged orders) or lowly heritable (lower left, 40 “other” merged orders) fractions of the dataset are presented within the dashed lines, as their Log_2_ heritability ratios are undefined numbers.

To explore whether microbes with high heritability in the sorghum dataset are phylogenetically clustered, we partitioned the 1189 OTUs into highly heritable (*n* = 347) and lowly heritable fractions (*n* = 842) using an *H*^2^ cutoff score of 0.15 (Fig. 2a, Supplemental Table 5). Several bacterial orders, including verrucomicrobiales, flavobacteriales, and planctomycetales, were observed to have significantly greater numbers of OTUs that are highly heritable, as compared to the lowly heritable OTU fraction (Fisher’s exact test, *q* < 0.05, Fig. 2a, Supplemental Table 5). Notably, all 6 Flavobacteriales OTUs were only present in the highly heritable fraction (Fig. 2b); by contrast, 40 other bacterial orders were only observed within the lowly heritable fraction. Having established that some bacterial lineages had a higher proportion of OTUs that were highly heritable, we aimed to determine what fraction of the total read count abundance these heritable OTUs represented. In general, we observed that read count abundance per taxa correlated with heritability, with some exceptions (Fig. 2b). For example, Bacillalles, contained a smaller number of OTUs in the highly heritable than a lowly heritable fraction, but the percentage of reading counts attributable to its highly heritable OTUs was approximately eight-fold greater than those in the lowly heritable fraction, suggesting that its highly heritable members are abundant organisms within the rhizosphere (Fig. 2b). Collectively, these data imply that a specific subset of bacterial lineages is enriched for members susceptible to host genotypic selection.

**Fig. 3:**
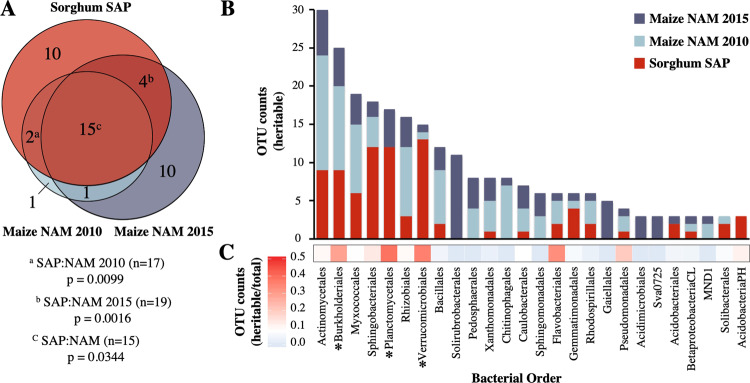
Heritability of rhizosphere microbes is conserved across maize and sorghum. **A** Proportional Venn diagram of bacterial orders containing highly heritable OTUs identified in this study (Sorghum SAP), compared with the heritable orders reported in a large-scale field study of maize nested association mapping (NAM) parental lines grown over two separate years, published in Walters et al. [5]. The top 100 heritable OTUs (based on *H*^2^) from each dataset were classified at the taxonomic rank of order to generate the Venn diagram. NAM highly heritable orders only present in the SAP lowly heritable fraction are represented by the blue sections. Superscript letters indicate the frequency that a random subsampling of 100 sorghum OTUs from the total 1189 sorghum OTUs (10,000 permutations) produced greater order-level overlap with maize OTUs from either single year (a/b) or both (c). **B** Stacked barplot displaying cumulative counts (*y*-axis) of OTUs identified as highly heritable in any of the three datasets for all bacterial orders (*x*-axis) which have a total of at least three highly heritable OTUs in an order. **C** The fraction of highly heritable sorghum OTUs relative to all sorghum OTUs within each order is displayed as a heatmap. Asterisks indicate orders enriched in highly heritable OTUs (Fisher’s exact test, *q* < 0.05).

Next, we hypothesized that despite the considerable evolutionary distance between maize and sorghum (two members of the grass family *Poaceae* that diverged more than 11 million years ago [48]), the bacterial lineages containing OTUs most responsive to host genotypic effects in maize would likely also contain OTUs exhibiting such susceptibility within sorghum. To test this, we compared the top 100 most heritable OTUs from both maize datasets (referred to as NAM 2010 and NAM 2015) and the sorghum dataset described above, resulting in a combined dataset of 300 OTUs spanning 65 bacterial orders. After removing bacterial orders not observed in the sorghum dataset (*n* = 18), we noted that more than half were observed in at least two of the datasets, and approximately one-third (*n* = 15) contained highly heritable OTUs in all three datasets (Fig. 3a). To determine if this overlap was significantly greater than is expected by chance, we performed permutational resampling of 10,000 sets of randomly chosen sorghum OTUs for comparison. Notably, we found that the overlap between the highly heritable sorghum fraction with both the individual maize heritable fractions and the combined heritable maize OTUs to be significant, compared with the resampled sorghum OTUs (NAM 2010 *n* = 17, *p* = 0.0099, NAM 2015 *n* = 19, *p* = 0.0016, combined *n* = 15, *p* = 0.0344) (Fig. 3a). Collectively, these results imply that there is conservation between the bacterial orders most sensitive to genotype across both maize and sorghum.

In an effort to identify the bacterial lineages with the greatest propensity for high heritability, we calculated the number of highly heritable OTUs in each of the shared highly heritable bacterial orders identified above. We noted that among bacterial orders containing the greatest number of highly heritable OTUs across all three datasets were several that represent large lineages frequently observed within the root microbiome; (e.g., actinomycetales) (Fig. 3b). We hypothesized that this result is likely driven in part by the overall frequency of these lineages within the rhizosphere microbiome, with more common lineages resulting in a greater fraction of highly heritable microbes due to their ubiquity. To help account for this, we normalized the frequency of highly heritable sorghum OTUs (*n* = 100) by total sorghum OTU counts (*n* = 1189) belonging to each order (Fig. 3c, Supplemental Table 6). These results demonstrate that while the prevalence of actinomycetales and myxococcales among highly heritable microbes is consistent with their general prevalence in the overall dataset, Burkholderiales and two other lineages, including the verrucomicrobia and planctomycetes, exhibited a significant enrichment (Fisher’s exact test, *q* < 0.05) in the highly heritable fraction not expected to be influenced by abundance alone.

### Genome-wide association reveals genetic loci correlated with rhizosphere microbial abundance

**Fig. 4:**
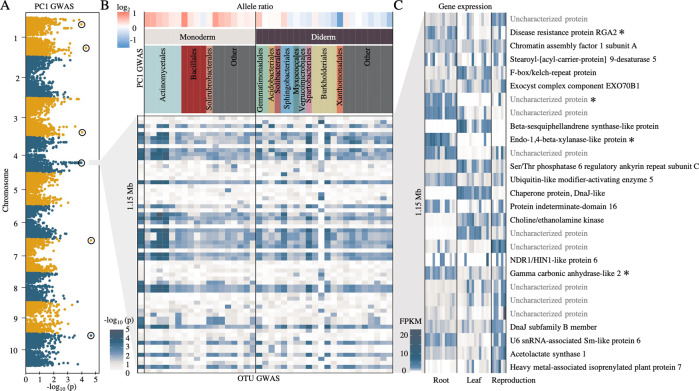
A sorghum genetic locus is correlated with rhizosphere microbial abundance. **A** Manhattan plot of PC1 community analysis GWAS. Top candidate SNPs above a threshold of –log_10_ (*p* = 10^–4^) are circled. **B** Individual OTU GWAS of all OTUs with at least 5 SNPs above a threshold of –log_10_ (*p* = 10^–2.5^) in the 1.15 Mb window identified on the same chromosome 4 locus identified by PC1 GWAS (lower heatmap). For each OTU, the log_2_ fold change in abundance between the sorghum major (red) or minor (blue) allele groups within this locus was determined (upper heat map). OTUs were grouped based on the predicted presence of one or two membranes (monoderm or diderm) within each bacterial order and colored as in Fig. 2. **C** Tissue-specific gene expression data for sorghum genes within the chromosome 4 locus. Darker blue indicates higher expression (normalized FPKM). Asterisks indicate genes whose expression is predicted to be root-specific.

Recent work in the leaf microbiome has demonstrated the potential utility of GWAS for uncovering host loci correlated with microbiome composition [18]. Here, we sought to use GWAS with rhizosphere microbiome datasets using both global properties of the OTU dataset and the abundances of individual OTUs. For overall community composition, a subset of PCs was selected from an analysis of the abundance patterns of the 1189 OTUs. To prioritize individual PCs for inclusion in our GWAS analysis, we determined the heritability scores of each of the top ten PCs, which explained 75% of the total variance in our dataset (Supplemental Fig. 5a). PCs with *H*^2^ equal to or greater than 0.25 (PC1, PC3, PC5, PC9, and PC10, Supplemental Fig. 5a) were subjected to GWAS (Supplemental Fig. 5b). We initially applied a strict Bonferroni-based threshold (adjusted *p*-value < 0.05/21,236) to our GWAS. However, no significant SNPs were identified using this method, suggesting this threshold was too stringent and masking potential true positive associations, due to a combination of small sample size (*n* = 200) and relatively low heritability (*H*^2^ = 0.35 for PC1) in our data. Despite the common use of applying multiple testing corrections, including Bonferroni corrections, to GWAS to define significance cutoffs, it is understood that these cutoffs are overly conservative due to the assumption that every genetic variant tested is independent of the rest [49, 50]. To discover potential true associations that were missed by Bonferroni correction, we applied an anti-conservative false discovery rate cutoff of –log_10_ (*p* = 10^–4^) (Benjamini–Hochberg, *q* < 0.22) to generate a list of top candidate SNPs. The GWAS analysis performed for PC1, which explained 21% of the total variance and had the second-highest heritability (*H*^2^ = 0.35), revealed a correlation between community composition and a locus on chromosome 4 that was among these top candidates (*n* = 6) (Fig. 4a, Supplemental Fig. 6a).

The MAF of candidate SNPs at the chromosome 4 locus ranged from 0.021 to 0.036. We carried out LD analysis between the most significant SNP (leading SNP) and the SNPs around the region. Results revealed that SNPs exhibiting high LD with the leading SNP were physically close (<1.6 Mb) and showed relatively high association signals (Supplemental Fig. 6b). As SNP imputation leverages local LD information, a cluster of SNPs in LD may be caused by SNP imputation. However, SNP imputation tends to be poor for minor alleles because less data is available to impute compared with common alleles [51]. Therefore, the presence of multiple GWAS signals exhibiting different allele frequencies at the chromosome 4 locus suggests the leading SNP was less likely to be a statistical artifact. Using the chromosome 4 locus SNP data, we separated sorghum genotypes into two allele groups, the major allele containing 343 sorghum genotypes and the minor allele containing 14 genotypes, with six minor allele-containing lines present in our 200 line GWAS subset. Using these six minor allele-containing genotypes and a closely related major allele genotype for each minor allele genotype, we performed a CAP ordination of the rhizosphere microbiome, constrained by allele group. This separated the rhizospheres of genotypes belonging to major and minor allele groups into distinct clusters (Supplemental Fig. 6c).

Subsequent GWAS analyses were performed using the other heritable PCs, PC3, PC5, PC9, and PC10, as inputs (Supplemental Fig. 7). We did not observe any SNPs below the *q* < 0.22 threshold for these PCs. This low signal is likely in part because the microbiome community has a relatively low heritability, the traits are highly polygenic, and the genome scans involve a large number of statistical tests. However, there was an identifiable peak on chromosome 6 of PC5 (*p*-value<7 × 10–4) and PC10 (*p*-value<1 × 10–4) (Supplemental Fig. 5b). As PCs are derived from linear combinations of the abundance of individual OTUs within the dataset, it is unclear whether the correlations observed on chromosomes 4 and 6 are driven by one common or two different sets of microbial lineages. To address this, we performed separate GWAS analyses using the abundances of each single OTU in our dataset as input (Fig. 4b, Supplemental Fig. 5c). From these analyses, we identified two distinct sets of 39 and 10 OTUs with significant correlations with the loci on chromosomes 4 and 6, respectively, and only a single OTU belonging to the order Burkholderiales that was shared between the two loci (Supplemental Fig. 5c). This implies that different sorghum loci are associated with the abundance patterns of different groups of microbes.

To understand the relationship between the identified peak on chromosome 4 (Fig. 4a) and the bacterial taxa with similar GWAS correlations at this locus (Fig. 4b), we first sought to understand how relative abundance for these 40 OTUs varied across the sorghum panel. We observed that the majority of OTUs that were more prevalent in sorghum genotypes containing the major allele belonged to monoderm lineages, while the majority of OTUs more prevalent in the minor allele group belonged to diderm lineages (Fig. 4b), suggesting that host genetic mechanisms at this locus are interacting with basal bacterial traits.

To explore which host genetic mechanisms might be driving the correlations observed on Chromosome 4, we examined tissue-specific expression patterns from publicly available RNA-Seq datasets obtained from phytozome v12.1 (ref. [44]) for all 27 genes in the 1.15 Mb interval (Fig. 4c, Supplemental Table 7). Of these candidates, we observed compartment-specific expression patterns, including several annotated candidates exhibiting strong root-specific activity: gamma carbonic anhydrase-like 2, a putative beta-1,4 endoxylanase, and disease resistance protein RGA2 (Fig. 4c).

### Sorghum genotypic data can predict microbiome composition

**Fig. 5:**
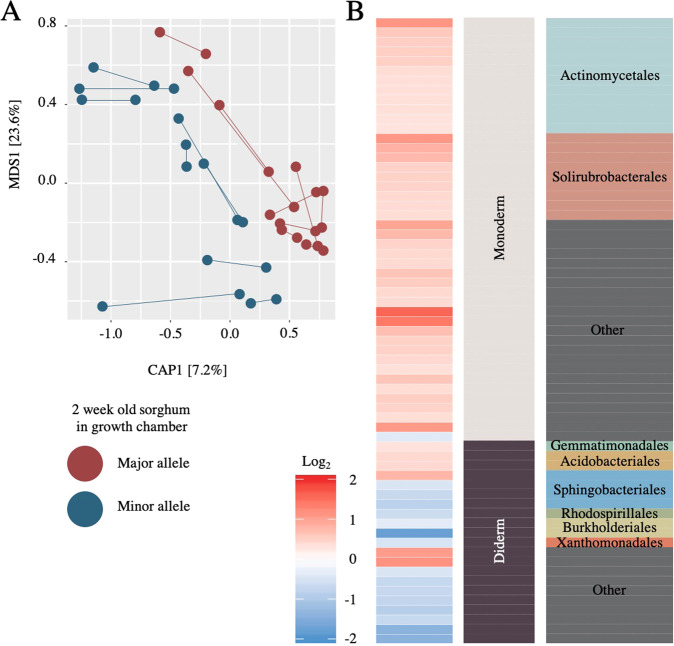
Sorghum genetic information can be used to predict rhizosphere microbiome composition under different growth conditions. **A** Canonical analysis of principal coordinates of the rhizosphere microbiome for nine major allele genotypes (red) and nine minor allele genotypes (blue) grown in a growth chamber for 2 weeks. Two replicates per genotype were used and are connected by lines. **B** For each indicator OTU, the log_2_ fold change in abundance between the sorghum major (red) or minor (blue) allele groups was determined. OTUs were grouped based on the predicted presence of one or two membranes (monoderm or diderm), within each bacterial order, and colored as in Figs. 2 and 4.

To validate that allelic variation at the candidate locus on chromosome 4 contributes to differences in rhizosphere composition, we conducted a follow-up growth chamber experiment with eighteen additional sorghum lines, including genotypes not present in the original study. To help disentangle phylogenetic-relatedness from locus-specific effects, we selected sorghum genotypes that spanned the diversity panel; additionally, for each minor allele genotype (*n* = 9), we included a phylogenetically related major allele line (*n* = 9) (Fig. 1a). Following two weeks of growth in a mixture of calcined clay and field soil in the growth chamber, we collected the rhizosphere microbiomes of each genotype and the microbiome composition was analyzed using 16S rRNA amplicon sequencing as in the main study. We conducted a regional association analysis using a mixed linear model that included a kinship matrix as the random effect. Using a stringent Bonferroni method, we detected several signals above the threshold that was in high LD with the original GWAS signal (Supplemental Fig. 8). A CAP ordination constrained on genotypic group separated the rhizospheres of genotypes belonging to major and minor allele groups into distinct clusters (Fig. 5a, PERMAnova *F* = 2.66, Df = 1, *p* = 0.0061), with genotype explaining approximately 7.5% (CAP1) of variance in the dataset.

To identify which taxa drive the clustering observed in our CAP analysis, and to compare this to taxa responsive to the chromosome 4 allele group in our main experiment, we performed an indicator species analysis on the validation dataset. A comparison of the significant indicator OTUs (*q* < 0.05) from each allele group in the validation dataset (*n* = 65) demonstrated similar trends in abundance of indicator OTUs as observed in the main experiment (Fig. 4b), with OTUs belonging to monoderm and diderm lineages enriched in the major and minor allele-containing lines, respectively. Interestingly, while most diderm lineages were more prevalent in the minor allele-containing lines, several diderm lineages including gemmatimonadales, acidobacteriales, and sphingobacteriales contained OTUs that were more abundant within major allele lines. Notably, this pattern was observed in the rhizospheres of 9-week old field-grown sorghum during the main experiment (Fig. 4b) and was also observed in 2 weeks growth chamber sorghum in the validation experiment (Fig. 5b). Collectively, this experiment supports the findings of our main experiment, in which allelic variation at a locus located on chromosome 4 was shown to correlate with the abundance of specific bacterial lineages.

## Discussion

### Host selection of plant rhizosphere microbiomes

Previous GWAS of plant-associated microbiome traits have often been conducted with leaf samples [16–18], and a recent GWAS was applied to root endophytes [19]. However, to our knowledge GWAS of the rhizosphere has not been attempted. In this study, we compared the overall correlation between host genotype and bacterial microbiome distances across leaf, root, and rhizosphere of *Sorghum bicolor*, and demonstrate that of the three, the rhizosphere represents the most promising compartment for conducting experiments to untangle the heritability of the sorghum microbiome. Notably, the degree of correlation between sorghum phylogenetic distance and microbiome distance was highest in the rhizosphere and lowest in the leaves. This greater correlation observed in the root and rhizosphere could be in part due to the phyllosphere’s relative compositional simplicity. Even *Arabidopsis* rosette leaves, which are in close proximity to the soil, harbor a distinct and relatively simple bacterial community compared to the root [19].

By contrast, the rhizosphere represents a highly diverse and populated subset of the soil microbiome and potentially offers a greater pool of microbes upon which the host may exert influence [52]. Alternatively, the rhizosphere’s greater correlation with microbiome composition could be caused by the plant’s relatively weaker ability to select epiphytes in its aboveground microbiome; while the arrival of phyllosphere colonists is largely thought to be driven by wind and rainfall dispersal [53], root exudation is known to control chemotaxis and other colonization activities of select members of the surrounding soil environment. This provides an additional mechanism for host selection of its microbial inhabitants prior to direct interaction with the plant surface [8, 54, 55]. Once in the root, microbes are subjected to additional selective pressures, including evading host immune systems [19, 56], that would determine if they persist as endophytes. While we observed the highest correlation sorghum phylogenetic distance and microbiome distance occurred in the rhizosphere, it is possible that other plant hosts may demonstrate the greatest selective influence within tissues other than the rhizosphere. Future efforts to investigate host control of the microbiome through GWAS or related techniques would benefit from a careful selection of sample types following pilot studies designed to explore heritability across different host tissues.

### Heritable rhizosphere microbes are phylogenetically clustered and similar across hosts

Within the rhizosphere, we demonstrate that microbiome constituents vary in *H*^2^, and highly heritable taxa show strong overlap with highly heritable lineages identified in maize, spanning fifteen different bacterial orders [5]. In particular, three of these orders, verrucomicrobiales, burkholderiales, and planctomycetales were significantly enriched in the highly heritable fraction of our dataset. As members of burkholderiales can form symbioses with both plant and animal hosts [57, 58], and some colonize specific members of a host genus or species [59], it is feasible that such strong relationships necessitated additional genetic discrimination between hosts. Within *Burkholderia* spp., this could be facilitated by their relatively large pan-genome, with diversity driven by large multi-replicon genomes and abundant genomic islands [60].

These observations suggest that evaluating bacterial heritability may identify new lineages for which close or symbiotic but previously undetected associations with plant hosts exist. For example, we observed several lineages with high heritability that are common in soil, yet prior evidence of plant-microbe interactions in the literature is lacking, including verrucomicrobiales and planctomycetales. Interestingly, high heritability in these lineages might be facilitated by the presence of a recently discovered shared bacterial microcompartment gene cluster present in both Planctomycetes and Verrucomicrobia, which confers the ability to degrade certain plant polysaccharides [61]. Indeed, microbiome composition is known to be driven in part by variations in polysaccharide-containing sources including plant cell wall components and root exudates [62]. Additional experimentation with bacterial mutants lacking this genetic cluster could be useful for revealing its role in shaping plant microbe interactions. Finally, we note that these results were generated in part through comparisons of datasets generated from two independent studies with different experimental designs and analysis pipelines; we anticipate that future experiments using a common garden approach could improve upon our efforts here to identify common heritable taxa across plant host lineages.

### Sorghum loci are responsible for controlling the rhizobiome

Our GWAS correlated host genetic loci and the abundance of specific bacteria within the host microbiome, as well as overall rhizosphere community structure. Our study builds upon previous research that applied GWAS to the Arabidopsis root microbiome [19], demonstrating that the use of this technique can be further expanded to the rhizosphere of a cereal crop plant. Using this method, we identified a locus on chromosome 4 that was correlated with specific bacterial lineages. Notably, we detected a similar association in a cross-validation experiment, which included both independent genotypes and different environmental conditions, providing strong support that this locus was a true positive, despite the modest statistical stringency applied to GWAS in this study. We observed several annotated candidate genes within the chromosome 4 locus exhibiting strong root-specific activity including gamma carbonic anhydrase-like 2, a putative beta-1,4 endoxylanase, and disease resistance protein RGA2. However, inferences of causal genes based on gene expression patterns come with significant limitations, as there is no requirement that a gene controlling this association would solely be expressed in roots. For example, architectural or hormonal changes in the plant phyllosphere could drive feed-forward effects on root exudate compositions [6, 9]. While our cross-validation experiment focused on a single locus on chromosome 4, plants are capable of influencing their microbiomes using a multitude of strategies, and many of these traits are predicted to be complex (i.e., controlled by multiple or many genes) [63]. As such, it is notable that we detected five additional candidate loci in the PC1 GWAS alone. In addition, a candidate locus on chromosome 6 was identified in both PC5 and PC10 community analysis GWAS. Strikingly, this locus was associated with a distinct set of microbes from the chromosome 4 locus identified by PC1 GWAS, suggesting that sorghum plants are able to use distinct mechanisms to modulate different groups of microbes. Future validation experiments using genetic mutants within these and other candidate genes can be used to help elucidate the underlying genetic element(s) responsible for the modulation of the rhizosphere microbiome.

## Conclusion

Although the underlying host genetic causes of shifts in the microbiome are not well understood, candidate-driven approaches have implicated disease resistance [6, 7], nutrient status [7, 64, 65], sugar signaling [66], and plant age [67, 68] as major factors. Non-candidate approaches to link host genetics and microbiome composition, such as GWAS, have the potential to discover novel mechanisms that can be added to this list. Here we show that GWAS can predict rhizosphere microbiome structure based on host genetic information, building on previous studies that have observed inter- and intra-species variation in microbiomes [1, 4, 5, 16, 19, 62, 69–71]. Collectively, our study adds to a growing list of evidence that genetic variation within plant host genomes modulates their associated microbiome. We anticipate that GWAS of plant microbiome association will promote a comprehensive understanding of the host molecular mechanisms underlying the assembly of microbiomes and facilitate breeding efforts to promote beneficial microbiomes and improve plant yield.

## Supplementary information

Supplemental Figures

Supplemental Tables

## Data Availability

All datasets and scripts for analysis are available through github (https://github.com/colemanderr-lab/Deng-2020) and all short-read data has been submitted to the NCBI and can be accessed through BioProject PRJNA612320.

## References

[1] Peiffer JA, Spor A, Koren O, Jin Z, Tringe SG, Dangl JL (2013). Diversity and heritability of the maize rhizosphere microbiome under field conditions. Proc Natl Acad Sci USA.

[2] Schlaeppi K, Dombrowski N, Oter RG, Ver Loren van Themaat E, Schulze-Lefert P (2014). Quantitative divergence of the bacterial root microbiota in Arabidopsis thaliana relatives. Proc Natl Acad Sci USA.

[3] Edwards J, Johnson C, Santos-Medellín C, Lurie E, Podishetty NK, Bhatnagar S (2015). Structure, variation, and assembly of the root-associated microbiomes of rice. Proc Natl Acad Sci USA.

[4] Naylor D, DeGraaf S, Purdom E, Coleman-Derr D (2017). Drought and host selection influence bacterial community dynamics in the grass root microbiome. ISME J.

[5] Walters WA, Jin Z, Youngblut N, Wallace JG, Sutter J, Zhang W (2018). Large-scale replicated field study of maize rhizosphere identifies heritable microbes. Proc Natl Acad Sci USA.

[6] Lebeis SL, Paredes SH, Lundberg DS, Breakfield N, Gehring J, McDonald M (2015). Salicylic acid modulates colonization of the root microbiome by specific bacterial taxa. Science.

[7] Castrillo G, Teixeira PJPL, Paredes SH, Law TF, de Lorenzo L, Feltcher ME (2017). Root microbiota drive direct integration of phosphate stress and immunity. Nature.

[8] Zhalnina K, Louie KB, Hao Z, Mansoori N, da Rocha UN, Shi S (2018). Dynamic root exudate chemistry and microbial substrate preferences drive patterns in rhizosphere microbial community assembly. Nat Microbiol.

[9] Saleem M, Law AD, Sahib MR, Pervaiz ZH, Zhang Q (2018). Impact of root system architecture on rhizosphere and root microbiome. Rhizosphere.

[10] Brachi B, Morris GP, Borevitz JO (2011). Genome-wide association studies in plants: the missing heritability is in the field. Genome Biol.

[11] Atwell S, Huang YS, Vilhjálmsson BJ, Willems G, Horton M, Li Y (2010). Genome-wide association study of 107 phenotypes in Arabidopsis thaliana inbred lines. Nature.

[12] Wu S, Tohge T, Cuadros-Inostroza Á, Tong H, Tenenboim H, Kooke R (2018). Mapping the Arabidopsis metabolic landscape by untargeted metabolomics at different environmental conditions. Mol Plant.

[13] Schaefer RJ, Michno J-M, Jeffers J, Hoekenga O, Dilkes B, Baxter I (2018). Integrating coexpression networks with GWAS to prioritize causal genes in maize. Plant Cell.

[14] Davenport ER, Cusanovich DA, Michelini K, Barreiro LB, Ober C, Gilad Y (2015). Genome-wide association studies of the human gut microbiota. PLoS ONE.

[15] Wang J, Thingholm LB, Skiecevičienė J, Rausch P, Kummen M, Hov JR (2016). Genome-wide association analysis identifies variation in vitamin D receptor and other host factors influencing the gut microbiota. Nat Genet.

[16] Horton MW, Bodenhausen N, Beilsmith K, Meng D, Muegge BD, Subramanian S (2014). Genome-wide association study of Arabidopsis thaliana leaf microbial community. Nat Commun.

[17] Wallace JG, Kremling KA, Kovar LL, Buckler ES (2018). Quantitative genetics of the maize leaf microbiome. Phytobiomes J.

[18] Roman-Reyna V, Pinili D, Borja FN, Quibod IL, Groen SC, Mulyaningsih ES, et al. The rice leaf microbiome has a conserved community structure controlled by complex host-microbe interactions. bioRxiv. 2019:615278. 10.1101/615278.

[19] Bergelson J, Mittelstrass J, Horton MW (2019). Characterizing both bacteria and fungi improves understanding of the Arabidopsis root microbiome. Sci Rep.

[20] Baerson SR, Dayan FE, Rimando AM, Nanayakkara NPD, Liu C-J, Schröder J (2008). A functional genomics investigation of allelochemical biosynthesis in Sorghum bicolor root hairs. J Biol Chem.

[21] Casa AM, Pressoir G, Brown PJ, Mitchell SE, Rooney WL, Tuinstra MR (2008). Community resources and strategies for association mapping in sorghum. Crop Sci.

[22] Morris GP, Ramu P, Deshpande SP, Hash CT, Shah T, Upadhyaya HD (2013). Population genomic and genome-wide association studies of agroclimatic traits in sorghum. Proc Natl Acad Sci USA.

[23] Pfeifer B, Wittelsbürger U, Ramos-Onsins SE, Lercher MJ (2014). PopGenome: an efficient Swiss army knife for population genomic analyses in R. Mol Biol Evol.

[24] Bradbury PJ, Zhang Z, Kroon DE, Casstevens TM, Ramdoss Y, Buckler ES (2007). TASSEL: software for association mapping of complex traits in diverse samples. Bioinformatics.

[25] Letunic I, Bork P (2019). Interactive tree of Life (iTOL) v4: recent updates and new developments. Nucleic Acids Res.

[26] Simmons T, Caddell DF, Deng S, Coleman-Derr D. Exploring the root microbiome: extracting bacterial community data from the soil, rhizosphere, and root endosphere. J Vis Exp. 2018:57561. 10.3791/57561. 10.3791/57561 PMC6101100 29782021

[27] Bolyen E, Rideout JR, Dillon MR, Bokulich NA, Abnet CC, Al-Ghalith GA (2019). Reproducible, interactive, scalable and extensible microbiome data science using QIIME 2. Nat Biotechnol.

[28] Magoč T, Salzberg SL (2011). FLASH: fast length adjustment of short reads to improve genome assemblies. Bioinformatics.

[29] Edgar RC (2013). UPARSE: highly accurate OTU sequences from microbial amplicon reads. Nat Methods.

[30] Wang Q, Garrity GM, Tiedje JM, Cole JR (2007). Naive Bayesian classifier for rapid assignment of rRNA sequences into the new bacterial taxonomy. Appl Environ Microbiol.

[31] Schloss PD, Gevers D, Westcott SL (2011). Reducing the effects of PCR amplification and sequencing artifacts on 16S rRNA-based studies. PLoS ONE.

[32] Paulson JN, Stine OC, Bravo HC, Pop M (2013). Differential abundance analysis for microbial marker-gene surveys. Nat Methods.

[33] Holland JB, Nyquist WE, Cervantes-Martínez CT. Estimating and interpreting heritability for plant breeding: an update. Plant Breed Rev. 2003; 22.

[34] Lee SH, van der Werf JH (2016). MTG2: an efficient algorithm for multivariate linear mixed model analysis based on genomic information. Bioinformatics.

[35] Rodríguez-Álvarez MX, Boer MP, van Eeuwijk FA, Eilers PHC (2018). Correcting for spatial heterogeneity in plant breeding experiments with P-splines. Spat Stat.

[36] Covarrubias-Pazaran G (2016). Genome-assisted prediction of quantitative traits using the R Package sommer. PLoS ONE.

[37] García-López R, Cornejo-Granados F, Lopez-Zavala AA, Sánchez-López F, Cota-Huízar A, Sotelo-Mundo RR, et al. Doing More with less: a comparison of 16s hypervariable regions in search of defining the shrimp microbiota. Microorganisms 2020;8:134. 10.3390/microorganisms8010134. 10.3390/microorganisms8010134 PMC7022540 31963525

[38] Kido T, Sikora-Wohlfeld W, Kawashima M, Kikuchi S, Kamatani N, Patwardhan A (2018). Are minor alleles more likely to be risk alleles?. BMC Med Genom.

[39] Yu J, Holland JB, McMullen MD, Buckler ES (2008). Genetic design and statistical power of nested association mapping in maize. Genetics.

[40] Zhou X, Stephens M (2012). Genome-wide efficient mixed-model analysis for association studies. Nat Genet.

[41] Oksanen J, Blanchet FG, Kindt R, Legendre P, Minchin PR, O’Hara RB, et al. Vegan: community ecology package software. 2016.

[42] Paradis E, Claude J, Strimmer K (2004). APE: analyses of phylogenetics and evolution in R language. Bioinformatics.

[43] Roberts DW, Roberts MDW Package ‘labdsv’. In: Ordination and multivariate. 2016.

[44] Goodstein DM, Shu S, Howson R, Neupane R, Hayes RD, Fazo J (2012). Phytozome: a comparative platform for green plant genomics. Nucleic Acids Res.

[45] Xu L, Naylor D, Dong Z, Simmons T, Pierroz G, Hixson KK (2018). Drought delays development of the sorghum root microbiome and enriches for monoderm bacteria. Proc Natl Acad Sci USA.

[46] Oberholster T, Vikram S, Cowan D, Valverde A (2018). Key microbial taxa in the rhizosphere of sorghum and sunflower grown in crop rotation. Sci Total Environ.

[47] Beilsmith K, Thoen MPM, Brachi B, Gloss AD, Khan MH, Bergelson J (2019). Genome-wide association studies on the phyllosphere microbiome: embracing complexity in host-microbe interactions. Plant J.

[48] Swigonova Z, Lai J, Ma J, Ramakrishna W, Llaca V, Bennetzen JL (2004). On the tetraploid origin of the maize genome. Comp Funct Genom.

[49] Perneger TV (1998). What’s wrong with Bonferroni adjustments. BMJ.

[50] Kaler AS, Purcell LC (2019). Estimation of a significance threshold for genome-wide association studies. BMC Genomics.

[51] Arouisse B, Korte A, van Eeuwijk F, Kruijer W (2020). Imputation of 3 million SNPs in the Arabidopsis regional mapping population. Plant J.

[52] Bodenhausen N, Horton MW, Bergelson J (2013). Bacterial communities associated with the leaves and the roots of Arabidopsis thaliana. PLoS ONE.

[53] Copeland JK, Yuan L, Layeghifard M, Wang PW, Guttman DS (2015). Seasonal community succession of the phyllosphere microbiome. Mol Plant Microbe Interact.

[54] Badri DV, Chaparro JM, Zhang R, Shen Q, Vivanco JM (2013). Application of natural blends of phytochemicals derived from the root exudates of Arabidopsis to the soil reveal that phenolic-related compounds predominantly modulate the soil microbiome. J Biol Chem.

[55] Zhang N, Wang D, Liu Y, Li S, Shen Q, Zhang R (2014). Effects of different plant root exudates and their organic acid components on chemotaxis, biofilm formation and colonization by beneficial rhizosphere-associated bacterial strains. Plant Soil.

[56] Chen T, Nomura K, Wang X, Sohrabi R, Xu J, Yao L, et al. A plant genetic network for preventing dysbiosis in the phyllosphere. Nature. 2020. 10.1038/s41586-020-2185-0 PMC7197412 32350464

[57] Angus AA, Agapakis CM, Fong S, Yerrapragada S, Estrada-de los Santos P, Yang P (2014). Plant-associated symbiotic Burkholderia species lack hallmark strategies required in mammalian pathogenesis. PLoS ONE.

[58] Kim JK, Lee BL (2015). Symbiotic factors in Burkholderia essential for establishing an association with the bean bug, Riptortus pedestris. Arch Insect Biochem Physiol.

[59] Shu L, Brock DA, Geist KS, Miller JW, Queller DC, Strassmann JE, et al. Symbiont location, host fitness, and possible coadaptation in a symbiosis between social amoebae and bacteria. Elife. 2018;7:e42660. 10.7554/eLife.42660. 10.7554/eLife.42660 PMC6336404 30596477

[60] Mannaa M, Park I, Seo Y-S. Genomic features and insights into the taxonomy, virulence, and benevolence of plant-associated burkholderia species. Int J Mol Sci. 2018;20:121. 10.3390/ijms20010121. 10.3390/ijms20010121 PMC6337347 30598000

[61] Erbilgin O, McDonald KL, Kerfeld CA (2014). Characterization of a planctomycetal organelle: a novel bacterial microcompartment for the aerobic degradation of plant saccharides. Appl Environ Microbiol.

[62] Bulgarelli D, Rott M, Schlaeppi K, Ver Loren van Themaat E, Ahmadinejad N, Assenza F (2012). Revealing structure and assembly cues for Arabidopsis root-inhabiting bacterial microbiota. Nature.

[63] Pascale A, Proietti S, Pantelides IS, Stringlis IA (2019). Modulation of the root microbiome by plant molecules: the basis for targeted disease suppression and plant growth promotion. Front Plant Sci.

[64] Khan GA, Vogiatzaki E, Glauser G, Poirier Y (2016). Phosphate deficiency induces the jasmonate pathway and enhances resistance to insect herbivory. Plant Physiol.

[65] Hiruma K, Gerlach N, Sacristán S, Nakano RT, Hacquard S, Kracher B (2016). Root endophyte colletotrichum tofieldiae confers plant fitness benefits that are phosphate status dependent. Cell.

[66] Yamada K, Saijo Y, Nakagami H, Takano Y (2016). Regulation of sugar transporter activity for antibacterial defense in Arabidopsis. Science.

[67] Wagner MR, Lundberg DS, Del Rio TG, Tringe SG, Dangl JL, Mitchell-Olds T (2016). Host genotype and age shape the leaf and root microbiomes of a wild perennial plant. Nat Commun.

[68] Edwards JA, Santos-Medellín CM, Liechty ZS, Nguyen B, Lurie E, Eason S (2018). Compositional shifts in root-associated bacterial and archaeal microbiota track the plant life cycle in field-grown rice. PLoS Biol.

[69] Lundberg DS, Lebeis SL, Paredes SH, Yourstone S, Gehring J, Malfatti S (2012). Defining the core Arabidopsis thaliana root microbiome. Nature.

[70] Haney CH, Samuel BS, Bush J, Ausubel FM. Associations with rhizosphere bacteria can confer an adaptive advantage to plants. Nat Plants. 2015;1:15051. 10.1038/nplants.2015.51. 10.1038/nplants.2015.51 PMC4806546 27019743

[71] Fitzpatrick CR, Copeland J, Wang PW, Guttman DS, Kotanen PM, Johnson MTJ (2018). Assembly and ecological function of the root microbiome across angiosperm plant species. Proc Natl Acad Sci USA.

