# Supplementary material for: Genome wide association study reveals plant loci controlling heritability of the rhizosphere microbiome
Source: ISME J. 2021 May 12;15(11):3181–94. doi: 10.1038/s41396-021-00993-z (PMC8528814; doi:10.1038/s41396-021-00993-z)
Supplement: Supplementary file 1 — Supplemental Figures [file 41396_2021_993_MOESM1_ESM.pdf]

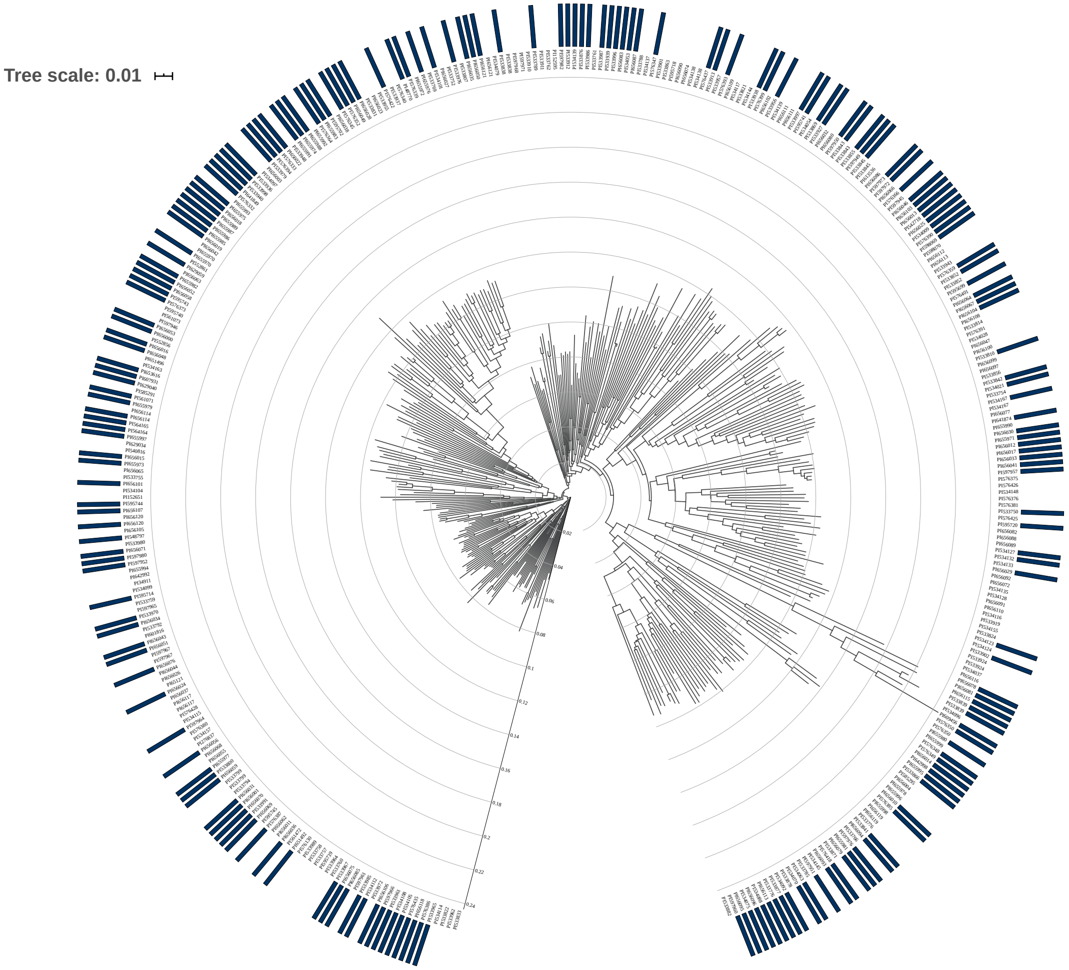

Supplemental Figure 1. Sorghum phylogenetic tree. Relationship between members of the 378 member sorghum association panel (inner ring) and the subset of 200 lines selected for GWAS (outer ring).

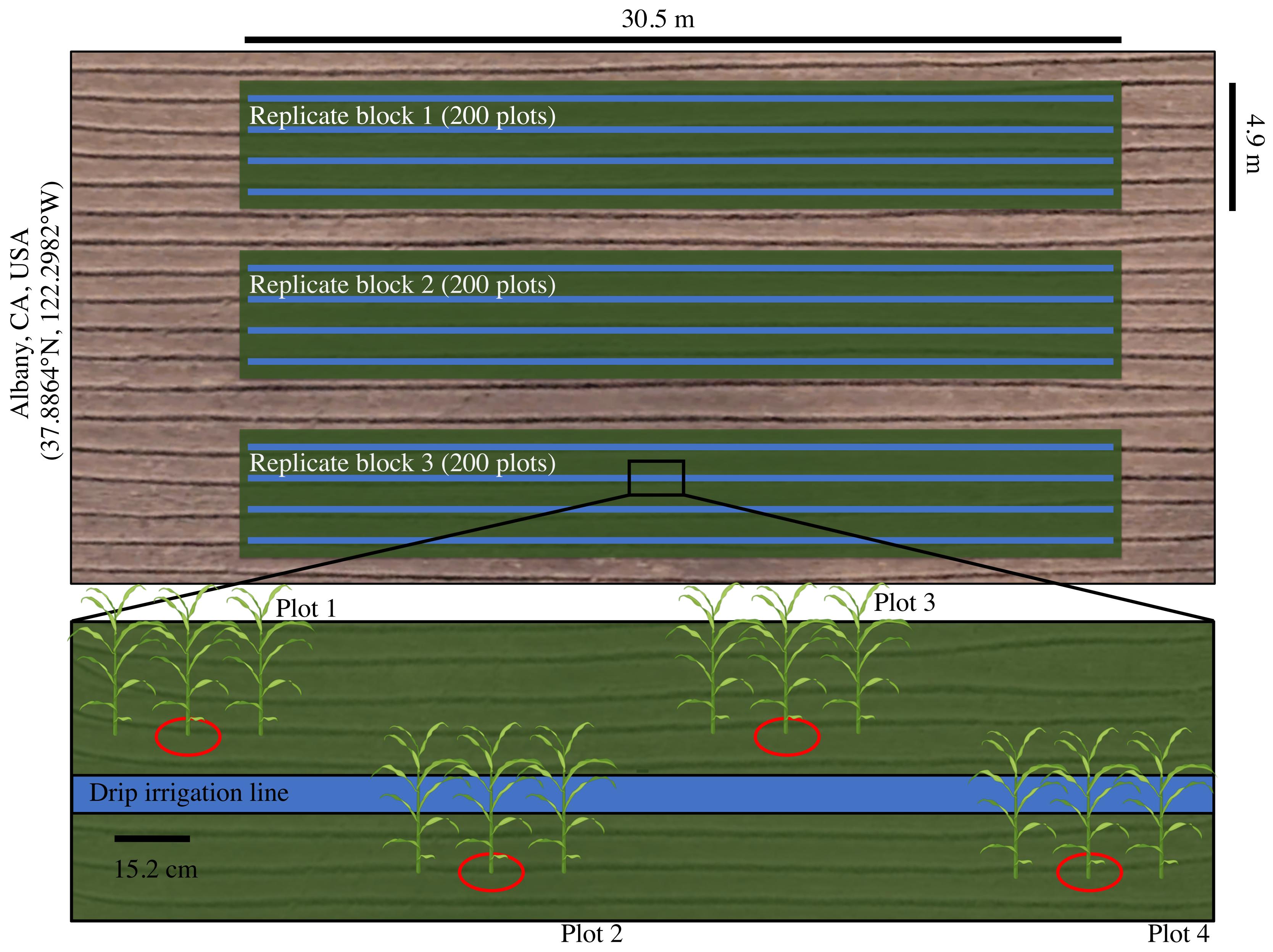

Supplemental Figure 2. Field experimental design. Three replicate blocks, each containing 200 plots. Each plot contains a different sorghum genotype with three replicate plants. Leaf, root, and rhizosphere from the center plant of each plot was harvested (Red circles). Sorghum plant models were created with Biorender.com.

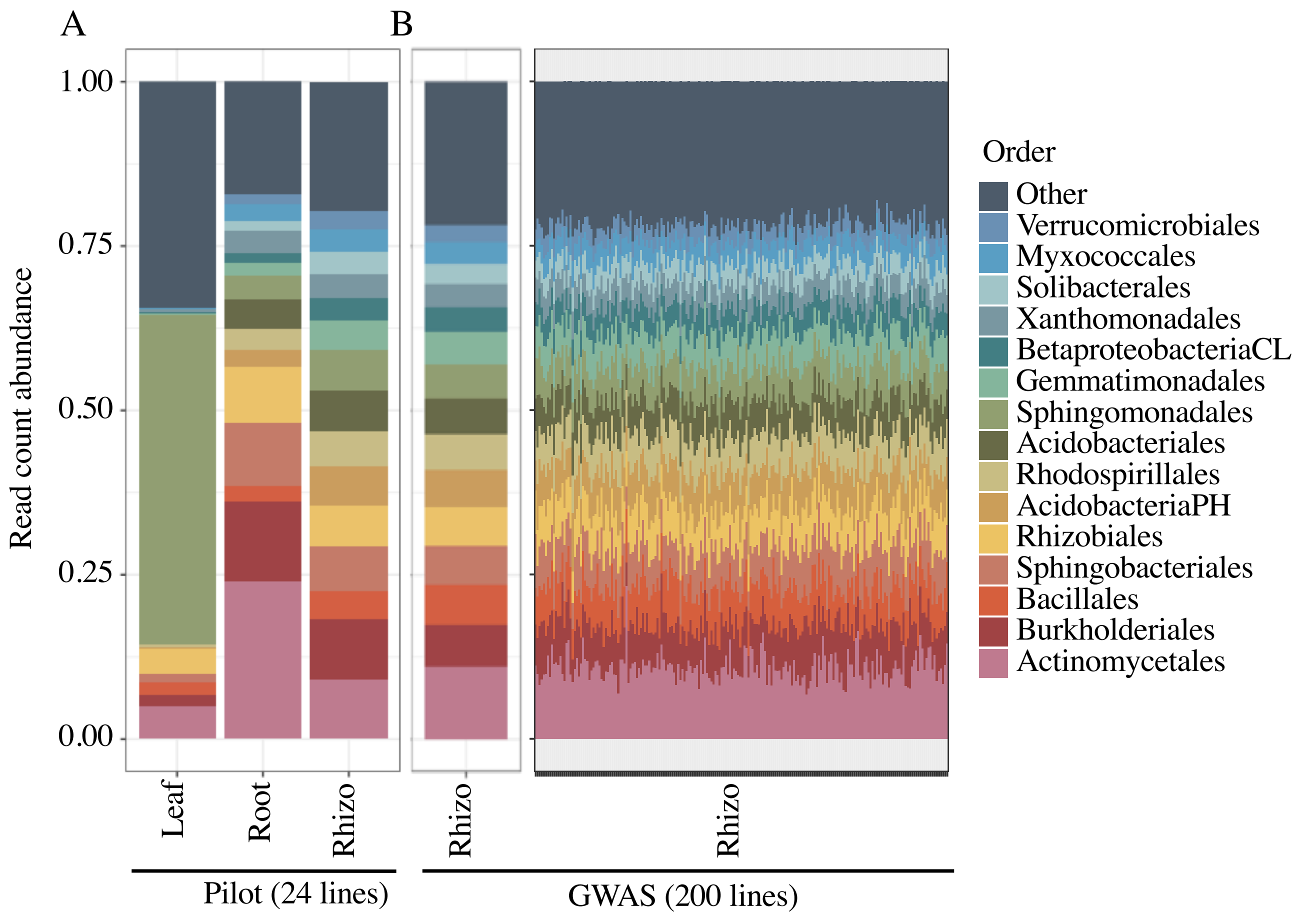

Supplemental Figure 3. Composition of the sorghum microbiome across sample types. A Order level relative abundances of sorghum leaf, root, and rhizosphere (rhizo) microbiomes used for GWAS sample type selection (Pilot). **B** Relative abundance of the top 15 orders in the 200 rhizosphere microbiomes used for GWAS, displayed both as an average relative abundance (left panel) and separated by individual sorghum line (right panel).

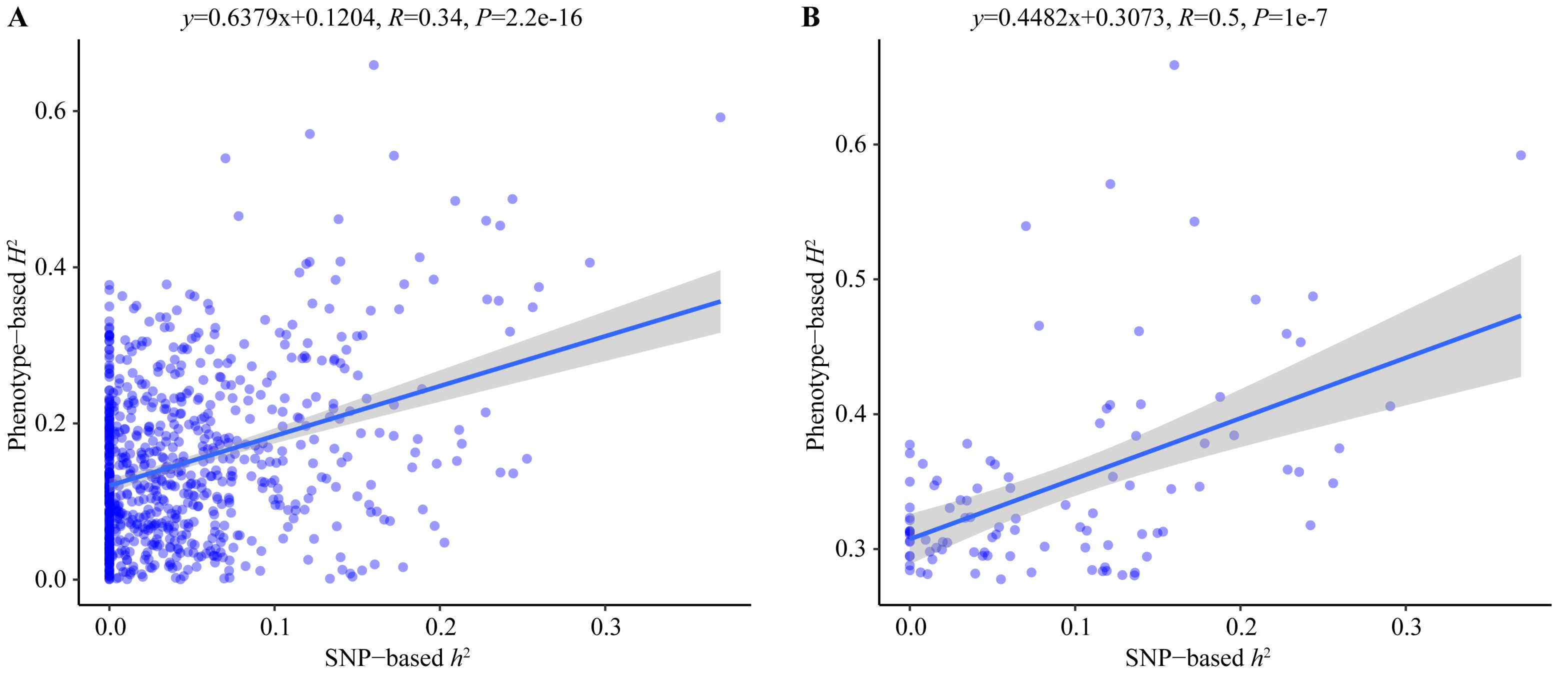

Supplemental Figure 4. Correlations between SNP-based h^2 and phenotype-based H^2. Performed for all OTUs (A) and the top 100 heritable OTUs (B). Blue lines denote the linear regression lines with shaded areas denoting the 95% confidence intervals.

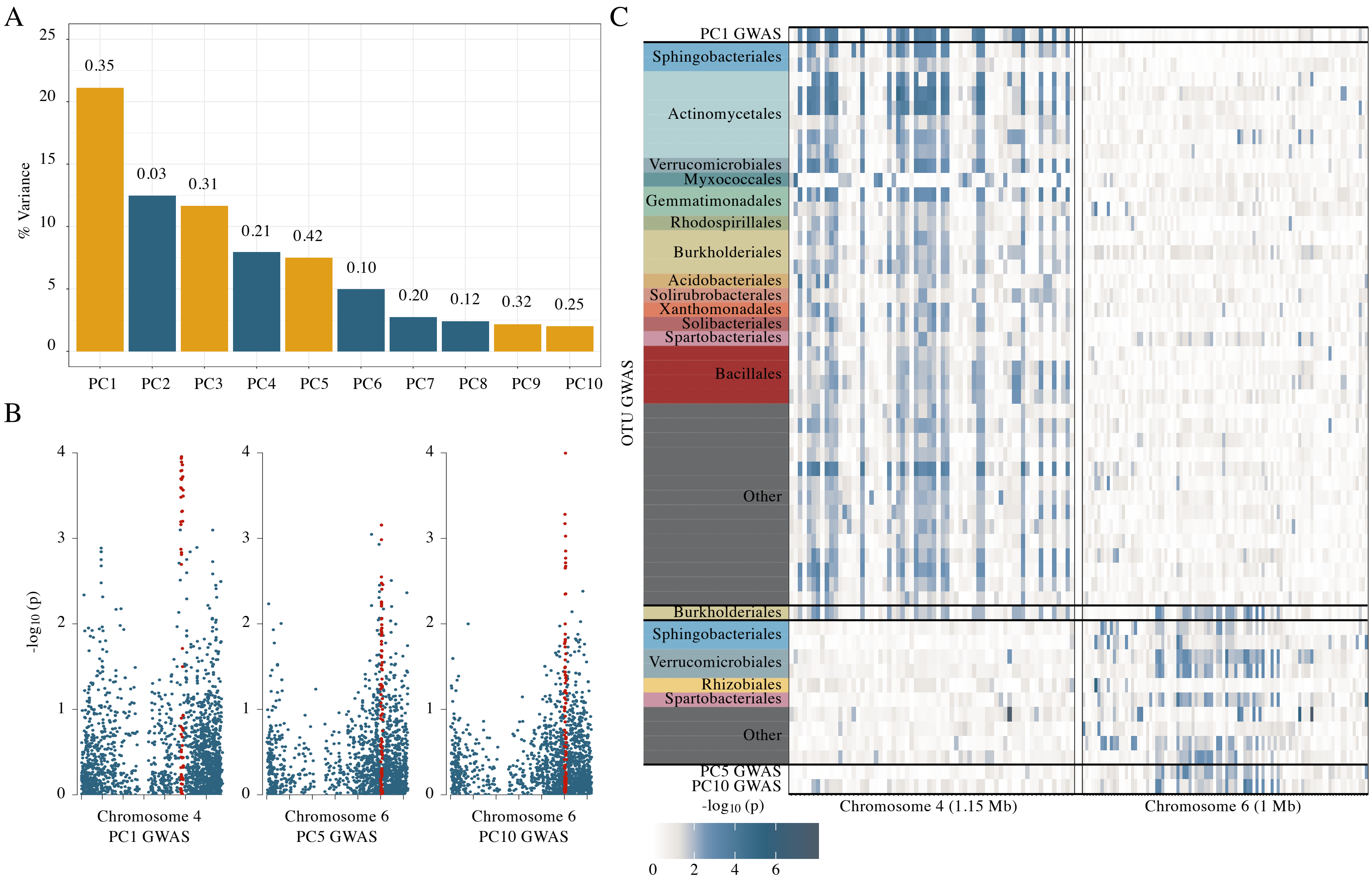

Supplemental Figure 5. The abundance of distinct sets of microbes are associated with different sorghum genetic loci. A Variance explained by each of the top 10 principal component axes. Numbers above the columns indicate the H^2 score associated with each PC. Yellow bars signify PCs with H^2 equal to or greater than 0.25. **B** Manhattan plots of PC1, PC5, and PC10 community analysis GWAS limited to the specific chromosomes with identifiable peaks. SNPs within the identified loci are colored red. **C** Individual OTU GWAS results for all OTUs with at least 5 SNPs above a threshold of $-\log_{10}$ ($p=10^{-2.5}$) in the 1.15 Mb window identified on the chromosome 4 locus identified by PC1 GWAS (top) or the 1 Mb window identified on the chromosome 6 locus identified by PC5 and PC10 GWAS (bottom). A single OTU belonging to the order Burkholderiales was identified by PC1, PC5, and PC10 GWAS (center). OTUs are grouped by the order they belong to and colored as in figure 2.

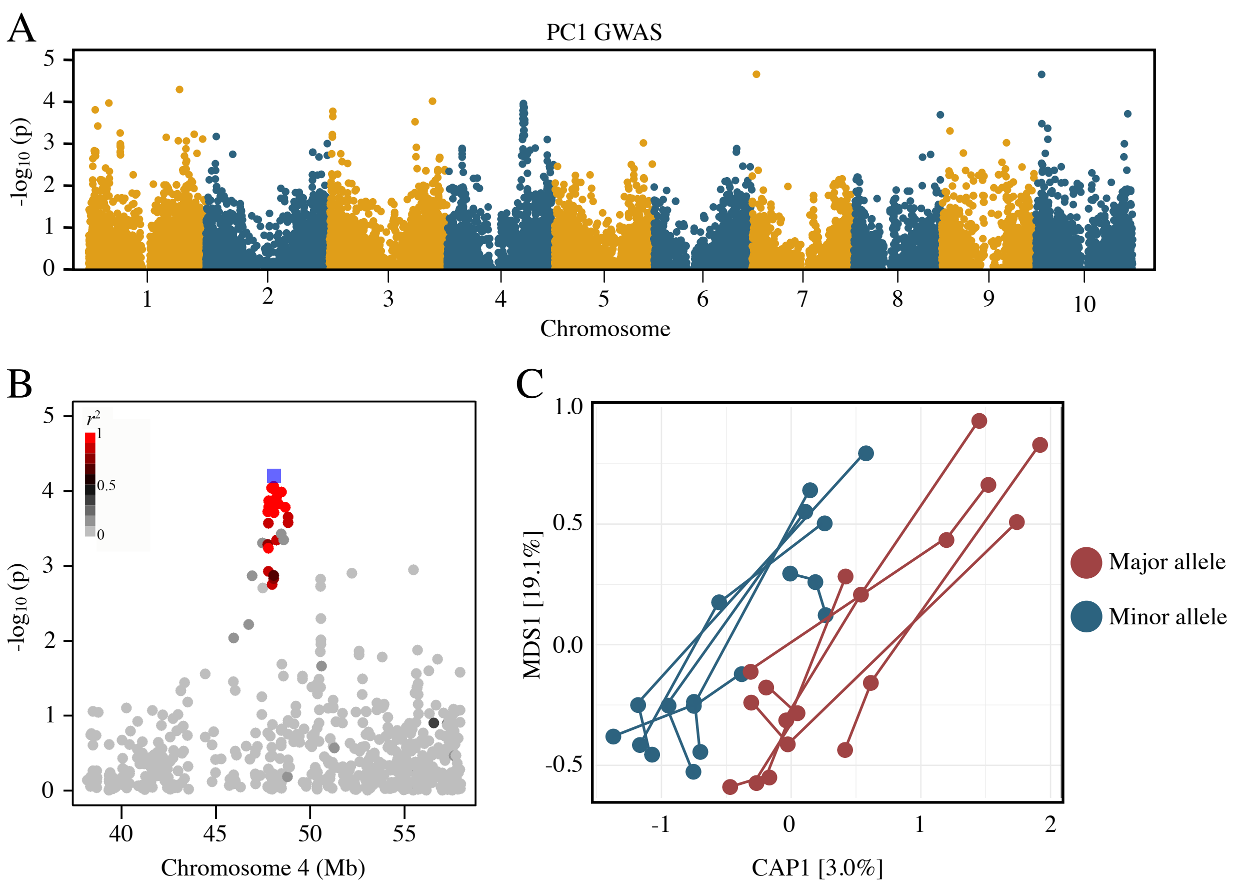

Supplemental Figure 6. Analysis of the chromosome 4 locus identified by PC1 GWAS. A Manhattan plot for the PC1 of community diversity GWAS. **B** LD analysis of the top candidate SNP on chromosome 4 (blue dot) and association signals located 10 Mb upstream or downstream from the most significantly associated SNP. Color coding of the dots denotes the LD values between SNPs in the region with the leading SNP. **C** Canonical Analysis of Principal Coordinates of the rhizosphere microbiome for the six minor allele containing genotypes (blue) and six closely related major allele genotypes (red) associated with the chromosome 4 locus identified by PC1 GWAS. Three biological replicates per genotype are connected by lines.

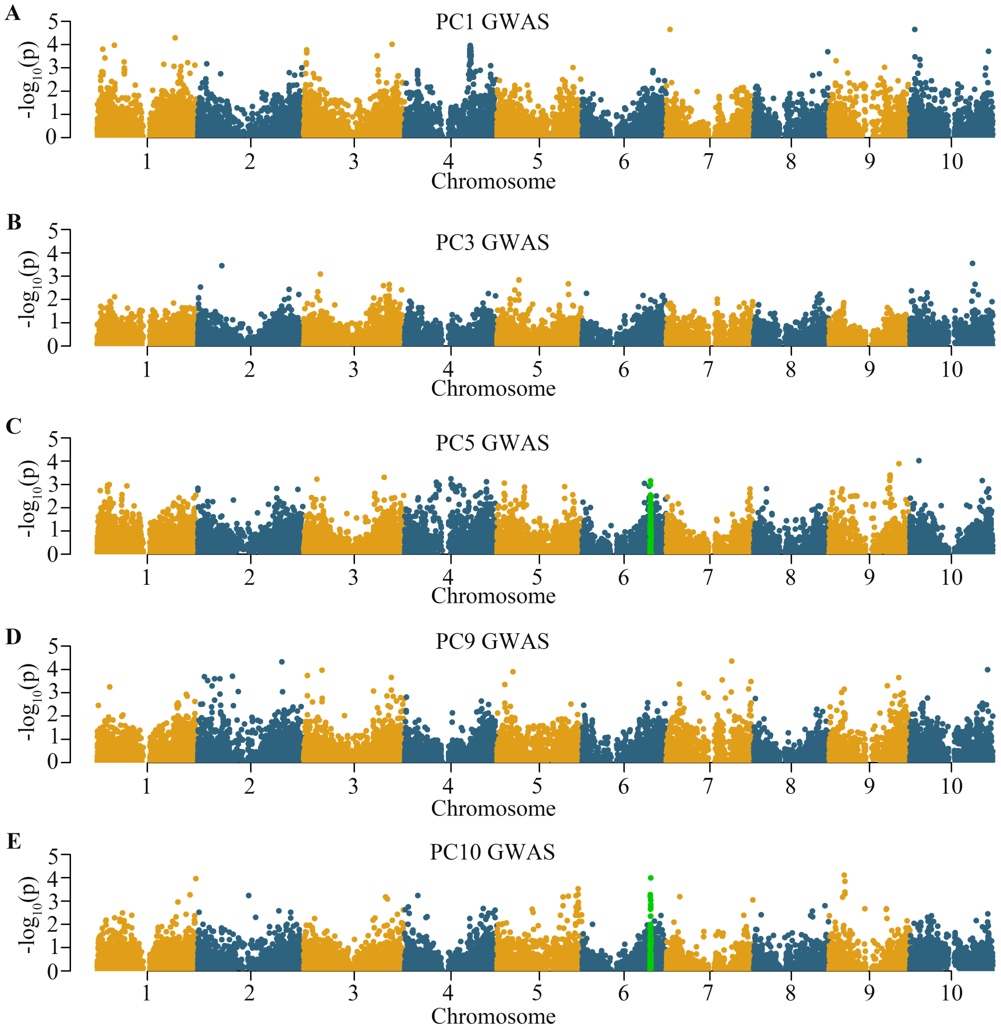

Supplemental Figure 7. GWAS analysis of all heritable PCs. A–E Manhattan plots for the PC1, PC3, PC5, PC9, and PC10 of community diversity GWAS. Green SNPs indicate a locus containing an identifiable peak on chromosome 6 of PC5 and PC10 GWAS.

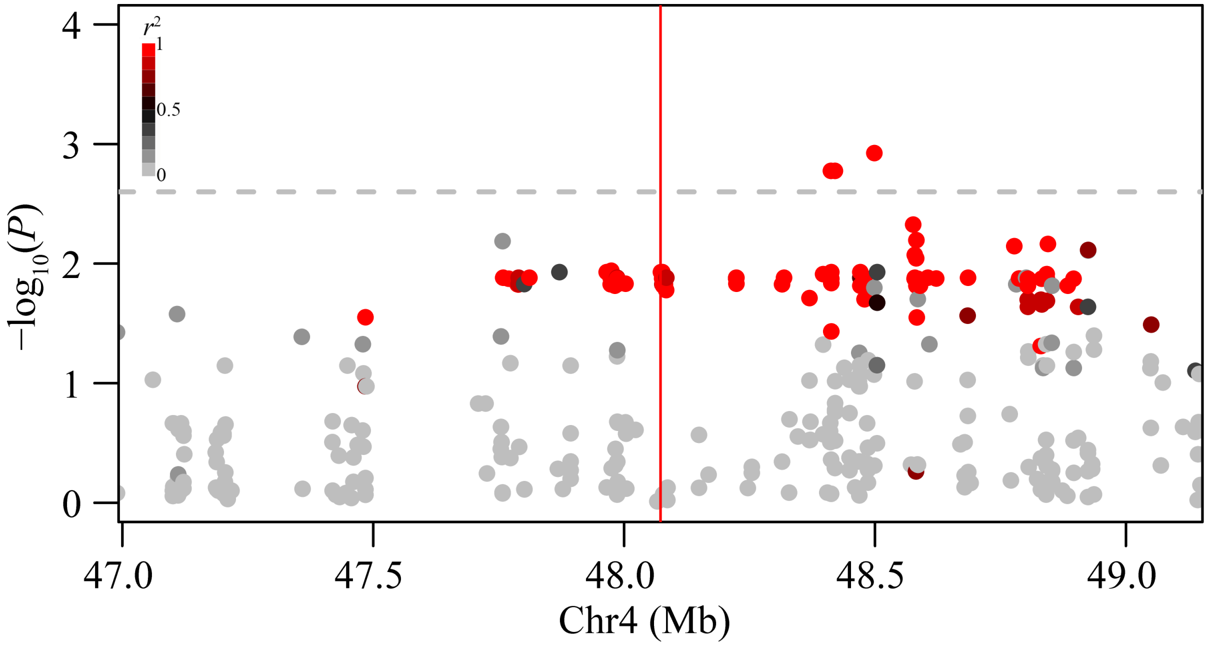

Supplemental Figure 8. Regional association analysis results for the first principle component of the microbiome community diversity. The red vertical line indicates the physical position of the leading SNP (S4_48072472) detected at chromosome 4 in the PC1 GWAS. The dashed horizontal line denotes the Bonferroni-adjusted significance threshold (p=2.5E-3). SNPs are color coded based on their LD (r^2) value with the leading SNP.
